# Peptide Nucleic Acids as a Tool for Site-Specific Gene Editing

**DOI:** 10.3390/molecules23030632

**Published:** 2018-03-11

**Authors:** Adele S. Ricciardi, Elias Quijano, Rachael Putman, W. Mark Saltzman, Peter M. Glazer

**Affiliations:** 1 Department of Biomedical Engineering, Yale University, New Haven, CT 06511, USA; adele.ricciardi@yale.edu (A.S.R.); rachael.putman@yale.edu (R.P.); mark.saltzman@yale.edu (W.M.S.); 2 Department of Genetics, Yale University School of Medicine, New Haven, CT 06520, USA; elias.quijano@yale.edu; 3 Department of Therapeutic Radiology, Yale University School of Medicine, New Haven, CT 06520, USA

**Keywords:** peptide nucleic acids, PNA, gene editing, nanoparticles, β-thalassemia, sickle cell disease, cystic fibrosis, *CCR5*, PLGA, Duchenne muscular dystrophy, triplex

## Abstract

Peptide nucleic acids (PNAs) can bind duplex DNA in a sequence-targeted manner, forming a triplex structure capable of inducing DNA repair and producing specific genome modifications. Since the first description of PNA-mediated gene editing in cell free extracts, PNAs have been used to successfully correct human disease-causing mutations in cell culture and in vivo in preclinical mouse models. Gene correction via PNAs has resulted in clinically-relevant functional protein restoration and disease improvement, with low off-target genome effects, indicating a strong therapeutic potential for PNAs in the treatment or cure of genetic disorders. This review discusses the progress that has been made in developing PNAs as an effective, targeted agent for gene editing, with an emphasis on recent in vivo, nanoparticle-based strategies.

## 1. Introduction

**Figure 1:**
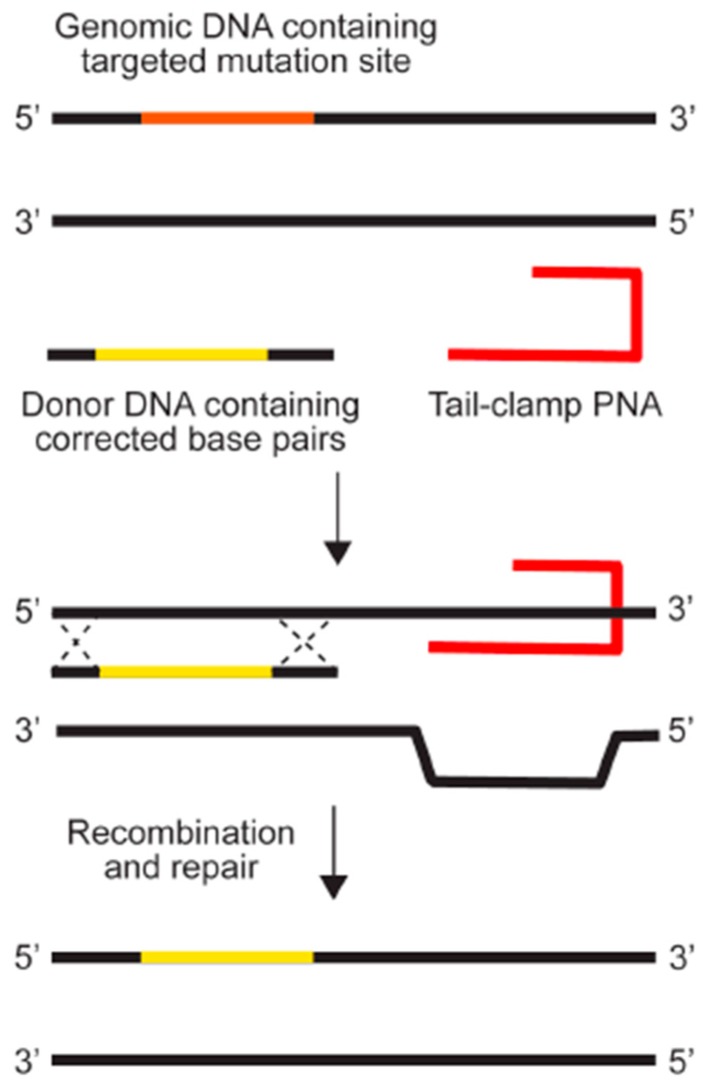
Genome modification using peptide nucleic acids. Reprinted with permission from the Yale Journal of Biology and Medicine [6].

Triple helical nucleic acid structures were first proposed by Linus Pauling prior to the establishment of the double-helical nature of DNA [1]. Biological formation of triplexes was first demonstrated four years later by Felsenfeld et al. in 1957 [2], and in the 60 years since then, substantial progress has been made in the application of triplex forming oligonucleotides (TFOs) as therapeutic molecules. Although both DNA and RNA can form triple-helix structures, one class of synthetic TFOs, peptide nucleic acids (PNAs), is particularly amenable for therapeutic application due to favorable biochemical and biophysical properties of the molecule. PNAs, which were first synthesized in 1991 by Peter E. Nielsen and colleagues [3], are synthetic DNA analogs in which the phosphodiester backbone has been replaced with *N*-(2-aminoethyl)-glycine units linked by peptide bonds. The charge neutral backbone allows PNA to bind to DNA or RNA via Watson-Crick hydrogen bonds, with binding affinities significantly higher than those of negatively-charged DNA oligomers [4]. PNAs are also highly stable and resistant to cleavage by proteases or nucleases [5]. These favorable biological properties have spurred many therapeutic applications for PNAs, such as inhibiting transcription, translation, or the activity of microRNAs, that have been previously reviewed [6]. PNAs have also recently been described as microRNA target protectors [7]. Further, the ability of PNAs to form triplexes with genomic DNA (gDNA) in a site-directed manner has also been harnessed as a strategy to achieve targeted gene editing at endogenous loci. PNAs are capable of binding with cognate DNA either along the major groove of dsDNA by Hoogsteen-base pairing at homopurine/pyrimidine stretches or binding directly by Watson-Crick base pairing after DNA duplex strand invasion [4]. Triplex formation creates an altered helical structure that is recognized by endogenous DNA repair factors that can induce recombination of a single-stranded donor DNA encoding a desired modification at a nearby genomic location (Figure 1) [8,9,10,11]. The ability of PNAs to induce recombination comes directly from their ability to tightly bind at their cognate sites, as they do not possess any direct nuclease activity, a feature that greatly increases the safety profile of PNAs.

**Figure 2:**
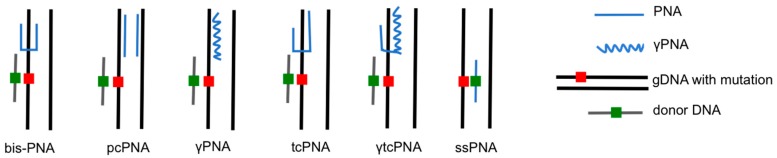
Examples of triplex structures used for PNA-mediated gene editing.

The earliest PNAs used for gene editing were designed to be dimeric, or “bis-PNAs”, in which two PNA strands of identical length are connected by a flexible linker [10]. Bis-PNAs bind by Watson-Crick and Hoogsteen base pairing along a single strand of homopurine DNA, forming a PNA/DNA/PNA triplex, while displacing the second strand of duplex DNA (Figure 2) [10,12,13,14,15]. Other PNA designs, such as pseudo-complementary PNAs (pcPNAs) have also been used to successfully achieve gene editing [16]. The most promising gene editing results, to date, have been achieved using a tail-clamp PNA (tcPNA) design in which the Watson-Crick binding domain is extended beyond the length of the Hoogsteen binding domain [17,18,19,20,21,22]. The so-called “tail” of a tcPNA protracts past the homopurine recognition site into mixed sequence DNA, which forms a stretch of duplex DNA in addition to the PNA/DNA/PNA triplex formed at the homopurine DNA target. The longer target site leads to enhanced binding specificity as well as a longer stretch of helical distortion. The success of tcPNAs has been further amplified with the introduction of chemical modifications, such as “miniPEG” substitutions on the PNA backbone [22].

The potential of these molecules to function as a therapeutic for systemic, human disease was realized though a fortuitous collaboration between the labs of Dr. Peter M. Glazer and Dr. W. Mark Saltzman of Yale University that demonstrated non-toxic and effective delivery of PNA and donor DNA using biodegradable nanoparticles [23]. This review discusses the advance of PNAs as a gene editing tool, as well as the development of biodegradable delivery systems primed for in vivo applications.

## 2. In Vitro Gene Editing

PNA induced gene correction was first demonstrated in the lab of Dr. Peter Glazer in 2002 by Rogers et al. using a bis-PNA and donor DNA in human cell-free extracts [10]. The authors showed that both a bis-PNA conjugated to a short donor DNA fragment and unconjugated bis-PNA and donor DNA are capable of mediating site-specific donor recombination in a *supFG1* reporter plasmid, with the unlinked reagents leading to a higher correction frequency. The plasmid contains an inactivating G to C mutation at position 144 in a *supFG1* reporter gene. Since *supFG1* encodes an amber suppressor tRNA, functional gene correction can be detected by plasmid transformation into bacteria containing an amber stop codon in the *lacZ* gene, with consequent β-galactosidase activity measured using a chromogenic substrate. Using this assay, gene correction by unlinked bis-PNA and donor DNA occurred at a frequency of 0.08% (Table 1). The donor DNA by itself was slightly active, leading to a low level of mutation reversion, results that are consistent with the ability of short DNA fragments to mediate recombination [24,25]. The effect of PNA and donor DNA treatment was over five-fold higher than the donor DNA alone, indicating the capacity of PNA to stimulate recombination between the target and donor DNA [10]. Though promising, these results were achieved in human cell-free extract, a system that circumvents the need for simultaneous intranuclear colocalization of the two molecules that would be necessary to achieve gene repair intracellularly.

In 2008, Chin et al. first studied intracellular PNA-mediated gene repair by co-transfecting bis-PNA and donor DNA into Chinese hamster ovary (CHO) cells containing a β-thalassemia causing human β-globin splice-site mutation [12]. In this reporter cell line, a single copy of GFP gene is interrupted by the second intron of the human β-globin gene containing a G to A splice site mutation (GFP/IVS2-1^G→A^). This mutation abrogates the normal donor splice site while activating a cryptic splice site, resulting in a GFP mRNA that retains an additional 47 nucleotides and produces no GFP protein expression. Thus, this system can be used as measure of gene editing, since correction of the splice-site mutation would result in GFP expression. The co-transfection by electroporation of bis-PNA and donor DNA to S-phase synchronized CHO-GFP/IVS2–1^G→A^ cells restored GFP expression in 0.2% of cells. Mutation correction was found to be dependent on cell cycle, with lower correction frequencies measured in asynchronous cells. The highest GFP expression was achieved with a PNA that bound to a homopurine sequence within the β-globin gene that was 193 base pairs from the mutation site. Lower levels of recombination were also measured using PNAs that were designed to bind to four additional homopurine sites ranging from 34 to 829 base pairs from the mutation of interest. GFP expression was observed using PNAs that targeted both the coding and noncoding DNA strand. This study used a donor DNA that corresponded to the coding strand, but PNA-mediated gene correction has also been observed with a donor DNA that corresponds to the noncoding strand [17,18,19,23]. Enhanced gene correction frequencies of >0.4% were detected when bis-PNA/donor DNA transfected cells were treated with chloroquine (Table 1), a lysomotropic agent that was previously reported to enhance PNA transfection into cells [26,27]. The enhancement measured after lysosomal disruption is attributed to the release of trapped nucleic acids, suggesting that the bioavailability of intracellular PNA and DNA is a critical factor contributing to correction efficiency. This work also demonstrated the persistence of PNA-mediated genomic correction—sequencing of sorted GFP-fluorescent cells that were maintained in culture for one month revealed the expected single base pair change at the genomic level [12].

In addition to targeted gene correction in the GFP-reporter construct, the authors demonstrated the efficacy of PNA-mediated gene modification in K562 cells, mouse bone marrow cells containing the human β-globin locus, and primary CD34^+^ progenitor cells. PNA-modified CD34^+^ cells were capable of differentiating into erythroid and myeloid lineages, in which the β-globin modification was still detectable [12]. These results in primary human cells support the feasibility of a transplantation based approach, using ex vivo PNA-mediated gene correction of mobilized peripheral blood progenitor cells in individuals with hematologic disorders.

In the previously described studies, PNA-mediated gene modification was achieved by designing bis-PNAs that bind to homopurine/homopyrimidine tracts, forming a PNA/DNA/PNA triplex (Figure 2). This strategy is limited by the requirement for a polypurine sequence in the target gene to enable triplex formation. To overcome this limitation, Lohse et al. designed pseudo-complementary PNAs (pcPNAs) that were capable of binding mixed purine-pyrimidine sequences to form two PNA/DNA duplexes (Figure 2) [28]. The pseudo-complementarity of pcPNAs is achieved with 2,6-diaminopurine and 2-thiouracil substitutions for adenines and thymines, respectively, in the PNA strands, rendering the two strands complementary, but incapable of binding each other due to steric hindrance. The base substitutions, however, do not prevent pcPNA binding to corresponding DNA sequences, rendering a pair of pcPNAs capable of prying open duplex DNA via formation of double-duplex invasion complexes. This PNA binding strategy has also been used for site-specific gene editing in the aforementioned CHO-GFP/IVS2–1^G→A^ cells. Co-transfection of a pair of pcPNAs targeting the β-globin intron 2 and a 51-mer donor DNA yielded correction of the splice-site mutation at a frequency of 0.01% [12]. The activity of the pcPNA was enhanced by modulating chromatin/DNA interactions with the histone deacetylase (HDAC) inhibitor, vorinostat (suberanilohydroxamic acid [SAHA]), to a frequency of 0.17%. A combination of S-phase synchronization and vorinostat treatment lead to the highest pcPNA and donor DNA modification frequency of 0.78% (Table 1) [16].

PNA and donor DNAs have also been used to modify additional, disease relevant genomic loci in vitro. Individuals with a naturally occurring 32 bp deletion in *CCR5*, a chemokine receptor required for intracellular HIV-1 entry, have been shown to be resistant to HIV-1 infection. In 2011, Schleifman et al. co-transfected PNA targeting the *CCR5* locus with a 60 nucleotide antisense donor DNA designed to introduce a stop codon, mimicking the delta32 mutation into THP-1 cells, a human acute monocytic leukemia line that expresses *CCR5* [17]. PNA/DNA treatment resulted in 2.46% targeted *CCR5* modification, which was shown to confer resistance to infection with R5-tropic HIV-1. In this study, “tail-clamp” PNA (tcPNA), a PNA with a Watson-Crick binding domain that extends beyond the homopurine sequence, was found to induce gene modification at a frequency higher than the 0.54% modification achieved with a conventional bis-PNA design, in which the Watson-Crick and Hoogsteen domains have equal length. The improved activity of tcPNA may be attributed to both increased PNA/DNA binding affinity and greater distortion of the underlying duplex target, leading to enhanced induction of DNA repair and recombination. As shown before, the induced genomic modification persisted as cells were continually passaged in culture for at least 98 days. Additionally, five corrected clones maintained in culture retained the targeted modification for 13 months, indicating the heritability of the modified allele [17].

To demonstrate the clinical applicability of this approach, Schleifman et al. additionally used tcPNA and donor DNA to modify *CCR5* in primary human CD34^+^ hematopoietic stem cells (HSCs), achieving editing frequencies of 2.8% (Table 1). PNA-modified CD34^+^ HSCs were next injected into immune-deficient NOD-*scid IL2rγ^null^* mice. Flow cytometry was used to confirm the presence of engrafted human HSCs four months after transplant and the presence of the targeted *CCR5* modification was confirmed using gDNA obtained from the spleen of engrafted mice. In a recent report, an HIV-1-positive patient with acute myeloid leukemia had no detectable viral load after transplantation with allogenic stem cells that were homozygous for the *CCR5*-delta32 mutation [29,30]. There is also evidence that individuals who are heterozygous for the mutation have a significantly reduced disease progression rate [31]. Thus, this work, in which *CCR5* is modified ex vivo with tcPNA and donor DNA, provides the basis for a therapeutic strategy in which transplantation of modified HSCs could offer a renewable source of virus-resistant immune cells.

In addition to mutation correction (β-globin) and gene disruption (*CCR5*), PNAs have also been used to create heritable nucleic acid changes that induce targeted gene expression. Hemoglobinopathies such as the β-thalassemias and sickle cell disease are caused by mutations that disrupt the normal function of the β-globin gene. The severity of these disorders can be diminished by a benign condition called hereditary persistence of fetal hemoglobin (HPFH), in which functional γ-globin—a globin subunit that is normally silenced—is expressed in adults. The majority of HPFH cases arise from mutations or deletions in the γ-globin promoter. Two mutations in the ^A^γ-globin gene promoter, −117 G→A and −175 T→C, are known to alter transcription factor binding, leading to decreased silencing of γ-globin expression. In 2013, Chin et al. used bis-PNA and a 100-nucleotide donor DNA to create the −117 mutation, as well as to introduce a hypoxia-responsive element (HRE) in CD34^+^ hematopoietic progenitor cells at a frequency of 1.63% (Table 1). The promoter modifications led to an increase in γ-globin expression that could be regulated by oxygen tension [32]. Although it is possible to correct single-base, disease-causing mutations, there are over 200 known mutations that lead to β-thalassemia. Correction of each mutation would require the design of mutation-specific reagents. Elevated fetal hemoglobin (HbF), however, reduces the severity of β-thalassemia and sickle cell disease, regardless of the causal mutation [33,34]. Thus, PNA-induced gene modification that reactivates γ-globin expression could be a treatment approach for all hemoglobinopathies.

## 3. Nanoparticle-Mediated PNA Delivery

**Figure 3:**
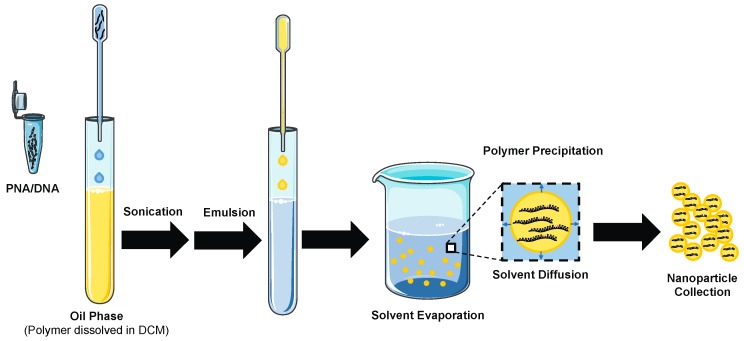
Fabrication of PNA/DNA polymeric nanoparticles using a double-emulsion solvent evaporation technique. Modified and reprinted with permission from the Yale Journal of Biology and Medicine [6].

For disorders affecting the hematologic system, ex vivo manipulation of target cell populations offers a viable treatment strategy, but for genetic disorders where ex vivo manipulation of target cell populations is challenging, such as cystic fibrosis or spinal muscular atrophy, the ability to directly modify cells in vivo is highly desirable. PNAs, however, do not cross the cellular membrane [36], so special intracellular delivery methods are required. Techniques for PNA delivery, such as electroporation, nucleofection, microinjection, conjugation to cell penetrating peptides, and conjugation to lipophilic moieties have all been successfully employed [26,37,38,39,40,41,42], but come with serious disadvantages. These approaches can be technically challenging, toxic to cells, rely on covalent modification of the PNA, decrease the activity of the PNA, or are inapplicable for use in vivo. Donor DNA must also be co-delivered with PNA for efficient gene modification, which further complicates the delivery of these agents. In 2011, collaboration between the labs of Dr. Peter M. Glazer and Dr. W. Mark Saltzman bore a novel approach: the use of biodegradable, polymeric nanoparticles (NPs) for PNA and donor DNA delivery [23]. McNeer et al. demonstrated that nanoparticles formulated from poly(lactic-co-glycolic acid) (PLGA)—a polymer that has been approved by the FDA in a variety of drug delivery applications—could be used for non-toxic and efficient delivery of PNA and donor DNA to primary human CD34^+^ progenitor cells, a cell type that is relatively more challenging to transfect than reporter cell lines [43]. PNA and donor DNA are encapsulated in NPs using a double emulsion solvent evaporation technique (Figure 3). PNA/DNA PLGA NPs are spheres with an average diameter of 150–250 nm and they carry a negative surface charge. Treatment of progenitor cells with bis-PNA and donor DNA NPs targeting the β-globin locus led to levels of site-specific gene modification up to 1% in a single treatment, without detectable loss in cell viability (Table 1). In contrast, nucleofected CD34^+^ cells were modified 60-fold less than the NP treated cells, with less than 10% cell viability. Treatment with donor DNA only NPs or with the donor DNA and PNA loaded into independent preparations of NPs also yielded genome modification. The highest levels of modification, however, were found when the PNA and DNA were co-loaded into the NPs. Genomic modification was also found to be dose dependent, with the highest levels of modification corresponding to the highest doses of NPs. PNA/DNA NP treatment did not affect the differentiation capacity of CD34^+^ cells into erythroid and neutrophil populations and as shown previously, differentiated cells maintained gene modifications [23].

McNeer et al. further demonstrated the generalizability of this approach by using PNA/DNA NPs to introduce a 6 bp mutation into the *CCR5* gene in human hematopoietic progenitor cells [19,23]. Schelifman et al. also used PNA/DNA NPs to modify *CCR5* in human peripheral blood mononuclear cells (PBMCs) at a 0.97% modification frequency (Table 1). The PNA-NP treated cells were engrafted into immunodeficient NOD-*scid* IL2rγ^null^ mice where the specific *CCR5* mutation was detected four weeks post-transplantation in splenic lymphocytes. After infection with R5-tropic HIV-1, the humanized mice with engrafted *CCR5*-NP treated PBMCs showed significantly higher levels of CD4^+^ T-cells and reduced viral loads compared with control mice engrafted with mock-treated PBMCs. In addition to pre-clinical efficacy, this work also examined the uptake and toxicity of NPs in PBMCs. The authors found that 24 h after the delivery of NPs encapsulating a fluorescent dye, nearly 100% of the PBMCs were associated with NPs, with the majority of the NPs localized intracellularly. In addition, NP treatment at the doses studied did not significantly affect cell viability nor induce the inflammatory cytokines TNF-α and IL-6 [19]. The ability to use PNA/DNA NPs to efficiently modify multiple disease-relevant sites in primary human progenitor cells in a non-toxic fashion strengthened the therapeutic potential for PNA-mediated gene editing and opened the door for future studies that explore the delivery of PNA and DNA oligomers in vivo.

## 4. In Vivo Gene Editing Using PNA-Nanoparticles

Polymeric nanoparticles containing PNA and donor DNA were first delivered in vivo via intravenous injection to modify the *CCR5* and β-globin genes in human hematopoietic stem cell engrafted NOD-*scid* IL2rγ^null^ mice [18]. PLGA nanoparticles were either unmodified or coated with a cell penetrating peptide derived from HIV-1 transactivating protein (TAT) or from the *Drosophila* antennapedia peptide (AP). Both TAT- and AP-modified PLGA NPs were found to increase donor DNA recombination when compared to unmodified particles in CD34^+^ cell in vitro. After intravenous treatment with unmodified or TAT-NPs loaded with PNA and donor DNA that targeted *CCR5* in mice engrafted with human hematopoietic cells, gene modification was detected in the bone marrow, spleen, thymus, small intestine, and lung [18]. This may indicate that PLGA NPs are distributed widely throughout the mouse before being taken up in human cells, allowing for gene modification. The use of NPs provided a significant advantage in vivo—only a low level of gene modification was detected when the mice were treated with naked PNA and donor DNA [18]. This result is consistent with previous studies in which naked oligos are rapidly cleared after intravenous injection [44]. Interestingly, unlike the in vitro results, the benefit of using TAT coated NPs over unmodified NPs was not conferred in vivo. Deep sequencing revealed 0.43% modification in the whole spleen (Table 1), but sequencing of individual colonies derived from human hematopoietic progenitors harvested from treated spleens, indicated that the modification of specific cell types, i.e., myeloid colony-forming cells, may be much higher (14–19%) [18].

As seen in vitro [23], intravenous PNA/DNA NP treatment did not affect the ability of mononuclear cells harvested from the bone marrow and spleen of treated mice to differentiate into myeloid and erythroid lineages. Serial transplant also confirmed that intravenous PNA/DNA NP treatment was capable of modifying HSCs in situ—when the bone marrow of PNA/DNA NP treated mice was transplanted into untreated mice, the targeted *CCR5* modification was detected in the bone marrow of the recipient mouse 10 weeks after transplantation. Additionally, the authors successfully showed the versatility of this approach by using intravenous PNA/DNA NP delivery to modify the human β-globin gene in the mice reconstituted with human hematopoietic cells and in an eGFP reporter mouse model. The work described above is the first demonstration of direct in vivo site-specific gene editing in human cells in a chimeric mouse and provides evidence that PNA-NPs can modify hematopoietic progenitor cells in vivo [18].

The next in vivo studies with PNA-NPs were focused on improving the design of the reagents used: both the design of the PNA and the polymer used for nanoparticle fabrication. The lab of Dr. Danith Ly of Carnegie Mellon designed a next generation PNA that contained diethylene glycol or “miniPEG” substitutions at the γ-position on the PNA backbone (γPNA). This gamma substitution increases PNA solubility and creates a chiral PNA that adopts a right-handed helical motif, a property that enhances binding affinity for the DNA target [5,45]. Next generation γPNAs can also invade mixed-sequence B-DNA, the most common form of double helical DNA, without homopurine sequence restrictions through Watson-Crick base pairing [46]. In 2014, Bahal et al. used a single-stranded γPNA co-encapsulated with a donor DNA in NPs to correct a disease causing β-thalassemia mutation both ex vivo and in vivo in a β-globin/eGFP reporter mouse. In this mouse, a mutation in nucleotide 654 of human β-globin intron 2 that creates an aberrant splice site is inserted within an eGFP transgene. The mutation causes retention of the intron, which abrogates eGFP fluorescence. Correction of the 654-mutation (T→C) restores proper splicing and allows for eGFP fluorescence, a phenotypic measure of gene editing. Relative to NPs containing an unmodified PNA, γPNA led to a four-fold increase in gene editing in β-globin/eGFP bone marrow cells ex vivo and in vivo [35].

The authors also investigated the use of NPs that contained a blend of a cationic polymer, poly(beta-amino ester) (PBAE) and PLGA. The cationic properties of this polymer allow for more efficient condensation of negatively charged oligonucleotides than PLGA. In addition, the tertiary amines present on the surface of PBAE NPs may buffer the low endosomal pH and allow for enhanced endosomal escape [47,48]. The Saltzman lab had previously shown that PBAE/PLGA blended NPs exhibited improved cellular uptake and plasmid transfection in cystic fibrosis bronchial epithelial cells (CFBEs) relative to PLGA NPs [49]. When used for gene editing, PBAE/PLGA NPs containing γPNA and donor DNA led to significantly higher gene editing frequencies in the β-globin/eGFP bone marrow cells than the frequency obtained with PLGA NPs [35].

Fields et al. further evaluated the in vivo potential of PBAE/PLGA blended NPs for gene editing in the lung [20]. Using an intranasal drug delivery route, blended PBAE/PLGA NPs showed increased cellular uptake and gene editing in the lung of the β-globin/eGFP mouse relative to PLGA NPs. Addition of a cell penetrating peptide, MPG, to the surface of the PBAE/PLGA NPs resulted in a substantial increase in NP uptake in gene editing in the total lung [20]. MPG, a synthetic peptide derived from the hydrophobic domain of the HIV gp41 fusion sequence and a hydrophilic domain from the nuclear localization sequence of the SV40 T-antigen [50], has previously been shown to improve the membrane permeability and nuclear trafficking of nucleic acids [51]. Enhanced PBAE/PLGA/MPG NP uptake and editing was also measured in alveolar macrophages and alveolar epithelial cells—two cell populations of interest for cystic fibrosis gene therapy.

In 2015, McNeer and Anandalingam et al. used tcPNA/DNA PBAE/PLGA/MPG NPs to correct the most prevalent cystic fibrosis transmembrane conductance regulator (CFTR) mutation, F508del [21]. Lack of CFTR function, a cAMP-dependent chloride channel, results in multi-organ disease, with the majority of symptoms affecting the respiratory system. The blended and surface modified NPs corrected 9.2% of CFTR mutations in human CFBE cells, which resulted in significant chloride efflux, indicating restored CFTR function. This NP formulation, containing mouse specific tcPNA and donor DNA, was then delivered intranasally to homozygous F508del mice. This treatment was well tolerated and intranasal NP delivery did not result in increases in inflammatory cytokines measured in bronchoalveolar lavage fluid, nor affect the histology of the lungs, which highlights the low immunogenicity of this approach. After four treatments, mouse CFTR correction in the nasal epithelium was assayed by measuring the nasal potential difference (NPD), a non-invasive method to detect chloride transport in vivo. Intranasal PBAE/PLGA/MPG NP treatment resulted in a significant decrease in the NPD, consistent with that of wild-type mice. Deep sequencing revealed 5.7% CFTR correction in the nasal epithelium and 1.2% in the lungs of NP treated mice (Table 1). Studies have indicated that as few as 6–10% of cells need to be corrected to reach normal levels of chloride ion transport in culture [52]. As designed, this system has the potential to achieve gene editing at clinically relevant levels.

PNA-NPs have also been used to produce functional disease improvement after intravenous delivery in a mouse model of human β-thalassemia. In this model, the two (cis) murine β-globin genes are replaced with a single copy of the human β-globin gene containing a β-thalassemia-associated splice site mutation in intron 2 at position 654. The mice produce reduced amounts of mouse β-globin chains and no human β-globin, resulting in β-thalassemia marked by microcytic anemia and splenomegaly. In 2016, Bahal et al. treated mice with β-thalassemia with PLGA NPs loaded with γtcPNA and donor DNA. The γPNA previously used for in vivo gene editing was a single-stranded PNA that does not form a triplex, but forms a PNA/DNA duplex and a displaced DNA strand [35]. Although this γPNA did provoke gene modification, it was previously shown that tcPNA, that forms a PNA/DNA/PNA triplex, is more efficient at invading dsDNA and does so with greater specificity [19]. Thus, this in vivo study aimed to make use of the favorable properties of both tail-clamp and γ-substitutions by using a γtcPNA. The authors found that γtcPNA/DNA NP treatment led to over 2-fold higher gene correction in bone marrow cells treated ex vivo than NPs that contained unmodified tcPNA and donor DNA. Intravenous delivery of the γtcPNA/DNA NPs after pretreatment with stem cell factor (SCF), the ligand for the c-Kit pathway, yielded levels of gene editing over 6% in sorted bone marrow HSCs (Lin- Sca1+ cKit+ CD150+ CD135-) in the β-thalassemia mouse. NP treatment ameliorated the disease phenotype, with elevation of hemoglobin levels into the normal range, reduced reticulocytosis, and reversal of splenomegaly. The elevation of hemoglobin was sustained throughout the duration of the study, up to 140 days post-treatment. Additionally, intravenous NP treatment did not lead to elevations in any inflammatory cytokines nor cause double stranded gDNA breaks, a measure of genotoxicity. These results, in which phenotypic disease improvement is achieved in vivo via non-toxic γtcPNA/DNA NP treatment, provide motivation for further development of PNA-NP mediated gene editing as a systemic treatment for human genetic disorders.

## 5. Gene Editing without a Donor DNA

Another approach to PNA-mediated gene editing that does not make use of donor DNA has been reported by Dr. Carmen Bertoni of the University of California, Los Angeles [53,54]. In her work on Duchenne muscular dystrophy (DMD), a disorder that is characterized by a complete absence of dystrophin protein, gene editing is achieved using single-stranded PNAs (ssPNAs) that bind over the genetic mutation of interest (Figure 2). The PNAs are either completely complementary or homologous to the coding strand of the target sequence, except for a single base mismatch aimed at correcting an A to T transversion in exon 10 of the dystrophin gene, a mutation that generates a cryptic splice at this position. Kayali et al. transfected ssPNA using Lipofectamine^TM^ 2000 into myoblasts derived from the *mdx^5cv^* mouse, a model of DMD [53]. Using quantitative PCR (qPCR) gene editing was estimated at ~3% for ssPNAs that targeted non-coding strand, with ~7% correction using ssPNAs that targeted coding strand. No correction was observed using control ssPNAs that are exactly the same as the PNAs used for correction, expect that they contain no mismatched bases and are completely complementary to the target gene mutation. The authors also reported that direct tibialis anterior injection of naked ssPNAs in *mdx^5cv^* mice resulted in dystrophin-positive fibers, as determined by dystrophin immunostaining, two weeks after treatment. qPCR analysis of gDNA isolated from treated muscles was used to estimate an in vivo correction efficiency of 2.8% for ssPNAs targeting the non-coding strand, and 3.3% for ssPNAs targeting the coding strand. Dystrophin-positive fibers were additionally detected four months after ssPNA treatment, indicating long-term persistence of the genetic correction [53]. In a follow up study by Bertoni’s group, Nik-Ahd et al. demonstrated that ssPNAs could be used to correct the dystrophin mutation in muscle satellite cells (SCs), the cells capable of self-renewing and differentiating into muscle fibers, from the *mdx^5cv^* mouse. Up to 2.1% of dystrophin gene correction was measured with qPCR analysis of gDNA isolated from SCs transfected with ssPNAs ex vivo. ssPNA treated SCs that were engrafted into the tibialias anterior of *mdx*/nude mice. Treated mice exhibited dystrophin expression for up to 24 weeks, when compared to mice engrafted with SCs that had been treated with control ssPNAs, which showed no dystrophin expression. Interestingly, the number of dystrophin-positive fibers was shown to significantly increase over time, resulting in a significant improvement in muscle morphology [54]. These results suggest that the PNA is not acting by stimulating site-specific recombination with a donor DNA, but is actually acting, by itself, as a source of genetic information to be used by a polymerase. It is yet to be demonstrated if a PNA can be a substrate for a polymerase, which merits further investigation. Additionally, this strategy may only be useful for the introduction of a single base-pair change into the genome, as it has been shown that greater than one mismatch in the PNA sequence can substantially reduce target sequence binding [55].

## 6. Off-Target Mutations and Genotoxicity

The generation of unintended, off-target mutations is of great concern with any gene editing therapy. The off-target mutation frequency associated with PNA-mediated gene editing has been measured as extremely low to undetectable (Table 2). Genomic changes induced by PNAs are a result of recombination that occurs at or near the site of triplex formation. Thus, the investigation into off-target mutations has focused on a targeted approach of sequencing loci partially homologous to the intended PNA binding site. It is highly improbable that off-target mutagenesis due to PNA binding would occur at any other genomic location—given the observed decreased binding affinity of PNA for mismatched or imperfect targets [17,55,56]. For example, Schleifman et al. have previously shown a four-fold decrease in PNA binding to the target site with a single base mismatch [17]. It is additionally unlikely that PNAs will cause spurious off-target mutations, provided that triplex structures do not induce double-strand DNA breaks (evidence discussed below). Consequently, an unbiased, genome-wide screen for off-target mutations after triplex-mediated gene editing has yet to be performed.

Unintended PNA-mediated off-target effects were first assayed in THP-1 cells after transfection of tcPNA and donor DNA targeting *CCR5*. The gene with the highest homology to *CCR5*, the *CCR2* gene, was sequenced in treated clones isolated by limiting dilution. Analysis of 1740 clones yielded no-off target changes, giving an upper limit to the off-target mutation rate of less than 0.057% [17]. Off-target mutations were next measured to be 0.004% in *CCR2* and undetectable in an additional partially homologous loci, *CCR4*, via deep sequencing after PNA and donor DNA delivery to PBMCs with polymeric nanoparticles. No evidence of genotoxicity was found after in vivo treatment with PNA/DNA NPs targeting *CCR5*—no mutations in *CCR2* were found in the 93 clones sequenced and deep sequencing revealed less than 0.004% off-target *CCR2* modification [18]. After treatment with CFTR targeting tcPNA/DNA NPs in vitro, no mutations were detected after the deep sequencing of a site partially homologous to the donor DNA and 13 sites partially homologous to the PNA. Additionally, no off-target mutations were detected after in vivo NP treatment in F508del mice [21]. Lastly, deep sequencing of six-partially homologous sites in gDNA derived from bone marrow cells after intravenous delivery γtcPNA/DNA NPs targeting the β-globin locus and ex vivo NP delivery to human CD34^+^ cells, led to extremely low levels of off-target mutations of 0.0032% and 0.000012%, respectively [22].

The off-target mutation rates seen with triplex-based gene editing are substantially lower than those reported after the use of nuclease-based strategies such as zinc-finger nucleases, TALENs, and CRISPR/Cas9. Nuclease-based strategies rely on the formation of double-strand gDNA breaks to incite DNA repair. Double-stranded breaks (DSBs) are likely to be repaired by the error-prone non-homologous end-joining (NHEJ) pathway, rather than by the higher fidelity homology directed repair (HDR) pathway. Bahal et al. demonstrated that PNA/DNA NP treatment did not result in detectable increases in DSBs in two assays for genotoxicity. By comparison, transfection of a Cas9 plasmid into primary fibroblasts resulted in significant formation of γH2AX foci, which are detected in nuclei by immune fluorescence and are markers of a chromatin modification that occur upon DSB formation [57]. The number of γH2AX foci per nucleus that were generated after Cas9 plasmid transfection was comparable to the number of foci formed in cells that were exposed to 5 Gy of ionizing radiation. The DSBs generated by Cas9 could be partially rescued with co-transfection of a guide RNA plasmid, but the level of DSBs was still significantly above background [22]. In a second assay for DSBs, electrophoresis of single lysed cells results in migration of fragmented DNA, which produces images that resemble comets when observed with fluorescence microscopy. The length of the comet “tail” corresponds to the number of DSBs in a cell. There was no detectable increase in comet tail moment of cells treated with PNA/DNA NPs, there was, however, a significant increase in comet tail moment after Cas9 plasmid transfection, indicating the presence of DSBs [21,22]. Despite the lower editing achieved with triplex-based gene editing than nuclease-based strategies, the relative lack of genotoxicity highlights PNAs as attractive therapeutic molecules, especially when considering translation to in vivo human therapies.

## 7. Mechanism of PNA Mediated Gene Editing

The ability of triplex-forming PNAs to stimulate recombination with donor DNAs has been shown to depend, in part, on the nucleotide excision repair (NER) pathway. NER is a versatile DNA damage-removal pathway that plays a critical role in repairing bulky DNA lesions [9,58]. Xeroderma pigmentosum group A (XPA) is an important NER factor responsible for recognizing altered DNA conformations and assembly of repair factors around the lesion [59]. The dependence on NER was first described in cell free extracts depleted of XPA, in which decreased recombination and repair were observed [10]. XPA siRNA knockdown in K562 cells also resulted in lower levels of bis-PNA induced gene modification [12]. The ability of pc-PNAs to stimulate recombination was also found to be dependent on XPA. In XPA-deficient and XPA-complemented human fibroblast cell lines, pcPNA and donor DNA were only effective in inducing gene modification at levels above background in the XPA-complemented line [16]. These findings support a hypothesis in which XPA plays a role in recognition PNA/DNA complexes that can provoke DNA metabolism to yield recombination. The mechanism of PNA-induced gene editing is an area of active investigation for the Glazer lab.

## 8. Measures to Enhance Gene Editing

The activity of PNAs and donor DNAs in gene editing has been enhanced in cells by methods that modify target site accessibility and improve oligomer delivery, including synchronization of cells in S-phase, treatment of cells with a histone deacetylase inhibitor, and the use of chloroquine, an endosome disrupting agent [12,16]. Gene editing was similarly enhanced ex vivo and in vivo with the use of SCF. In ex vivo treated bone marrow cells, SCF was found to enhance the gene editing efficiency of γtcPNA/DNA NPs from ~8% (without SCF) to ~15% (with SCF). This increase in editing is likely due to an increase in DNA repair gene expression. SCF treatment also yielded 5% gene correction in CD34^+^ hematopoietic progenitor cells after one treatment with γtcPNA/DNA NPs ex vivo. These cells were transplanted into NOD-*scid* IL2rγ^null^ mice, and gene correction of over 3% was detected eight weeks after transplantation. The effect of SCF on gene editing in the bone marrow raises the possibility that other cytokines, growth factors, or compounds that mobilize stem cells, could enhance gene editing in bone marrow and perhaps other tissues. Additionally, the mechanism by which SCF enhances gene editing may be independent of triplex formation and may also enhance the editing of nuclease-based techniques. Ultimately, a more comprehensive understanding of the mechanism of PNA-mediated gene editing will likely reveal key information that can be exploited to further enhance triplex-induced recombination.

## 9. Perspectives and Limits

Many factors affect gene correction frequency, such as the design of the PNA and donor DNA, the method of delivery of oligomers to the nucleus, the accessibility of the genomic target, and the cell cycle. These factors make some systems more amenable to editing and complicate the comparison of editing frequencies reported in other work.

For most studies, NP formulations are first tested in vitro, with the best performing in candidates used for in vivo studies. Given that in vitro test environments are extremely different than tissue microenvironments, it is likely that this approach may have limitations with regard to approximating in vivo efficacy [18].

Triplex formation appears to be safer than nuclease-based editing approaches [22]; the lack of toxicity and the much lower frequencies of off-target effects may offer the possibility for multiple treatments to achieve more significant effects.

## 10. Conclusions

PNAs have emerged as a powerful tool capable of inducing specific genomic changes at targeted locations. The studies highlighted in this review demonstrate editing of multiple disease-relevant targets both ex vivo and in vivo via the use of biodegradable, nanoparticle delivery systems. Advances in PNA design and nanoparticle formulation have led to levels of PNA-NP mediated gene editing that induce phenotypic correction in preclinical mouse models of β-thalassemia and cystic fibrosis. These recent successes in mouse models raise the possibility that PNA-NP mediated gene editing may eventually provide the basis for a new genetic therapy for single-gene hereditary disorders, such as sickle cell anemia, thalassemia, and cystic fibrosis.

## Figures and Tables

**Table 1:** PNA and donor DNA-mediated gene editing.

Drug Delivery System	Reagent	Study Design (Model, Target Gene)	Efficiency (Assay)	Year, Ref.
N/A	bis-PNA and donor DNA	human cell free extract (pSupFG1/G144C)	0.08% (β-galactosidase assay)	2002, [10]
electroporation or nucleofection	bis-PNA and donor DNA with chloroquine	cell culture (Chinese hamster ovary cells containing a human β-globin splice-site mutation, CHO-GFP/IVS2–1^G→A^ and human CD34^+^ progenitor cells)	0.4%—CHO cells (FACS)	2008, [12]
electroporation	pcPNA and donor DNA with SAHA	cell culture (Chinese hamster ovary cells containing a human β-globin splice-site mutation, CHO-GFP/IVS2–1^G→A^)	0.78% (FACS)	2009, [16]
PLGA NPs	bis-PNA and donor DNA	cell culture (human CD34^+^ cells, β-globin)	0.91% (limiting dilution, allele specific PCR)	2011, [23]
electroporation	tcPNA and donor DNA	cell culture (THP-1 and human CD34^+^ cells, *CCR5*)	2.8% (limiting dilution, allele specific PCR)	2011, [17]
electroporation	bis-PNA and donor DNA	cell culture (human CD34^+^ cells, γ-globin)	1.63% (allele specific qPCR)	2013, [32]
PLGA NPs	tcPNA and donor DNA or bis-PNA and donor DNA	in vivo (humanized NOD-*scid* IL2rγ^null^ mice, *CCR5* and β-globin)	0.43% (deep sequencing)	2013, [18]
PLGA NPs	tcPNA and donor DNA	ex vivo (human PBMCs engrafted into NOD-*scid* IL2rγ^null^ mice, *CCR5*)	0.97% (deep sequencing)	2013, [19]
PLGA NPs and PLGA/PBAE NPs, IV delivery	γPNA and donor DNA	in vivo (β-globin/eGFP transgenic mouse)	0.1% (deep sequencing)	2014, [35]
PLGA/PBAE/MPG NPs, intranasal delivery	tcPNA and donor DNA	in vivo (β-globin/eGFP transgenic mouse, intranasal delivery)	0.4% (FACS)	2015, [20]
PLGA/PBAE/MPG NPs, intranasal delivery	tcPNA and donor DNA	in vivo (F508del mice, CFTR, intranasal delivery)	5.7% (deep sequencing)	2015, [21]
PLGA NPs, IV delivery	γtcPNA and donor DNA with SCF	in vivo (IVS2-654 thalassemic mice, β-globin)	3.4% (deep sequencing)	2016, [22]

**Table 2:** On-target efficiency compared to off-target mutation rate.

Gene	Reagents	Source of gDNA	On-Target Modification Frequency	Off-Target Modification Frequency	Reference
*CCR5*	tcPNA/DNA	THP-1 cells	2.8%	<0.057%	[17]
*CCR5*	tcPNA/DNA PLGA NPs	human PBMCs	0.97%	0.004%	[19]
*CCR5*	tcPNA/DNA PLGA NPs	humanized NOD-*scid* IL2rγ^null^ mouse bone marrow	0.43%	0.004%	[18]
CFTR	tcPNA/DNA PBAE/PLGA/MPG NPs	CFBE cells	9.2%	<0.00001%	[21]
CFTR	tcPNA/DNA PBAE/PLGA/MPG NPs	mouse nasal epithelium	5.7%	<0.0001%	[21]
β-globin	γtcPNA/DNA PLGA NPs with SCF	total mouse bone marrow cells	3.9%	0.0032%	[22]
β-globin	γtcPNA/DNA PLGA NPs with SCF	human CD34^+^ cells	5.02%	0.000012%	[22]
